# Novel sensitive monoclonal antibody based competitive enzyme-linked immunosorbent assay for the detection of raw and processed bovine beta-casein

**DOI:** 10.1371/journal.pone.0182447

**Published:** 2017-07-31

**Authors:** Daniela S. Castillo, Alejandro Cassola

**Affiliations:** Instituto de Investigaciones Biotecnológicas—Instituto Tecnológico de Chascomús (IIB-INTECH), Universidad Nacional de San Martín (UNSAM)—Consejo Nacional de Investigaciones Científicas y Técnicas (CONICET), San Martín, Buenos Aires, Argentina; Instituto Butantan, BRAZIL

## Abstract

Cow milk protein allergy (CMPA) is the most common childhood food allergy, which can sometimes persist or can newly develop in adulthood with severe symptoms. CMPA's treatment is complete dietary avoidance of milk proteins. To achieve this task, patients have to be aware of milk proteins found as "hidden allergens" in food commodities. In regard to milk proteins, it has been reported that allergenicity of caseins remains unaffected upon heat treatment. For these reasons, we aimed to obtain monoclonal antibodies (mAbs) against native and denatured β-casein, one of the most abundant and antigenic caseins, in order to develop an indirect competitive ELISA (icELISA) to detect and quantify traces of this milk allergen in raw and processed foodstuffs. We developed two specific hybridoma clones, 1H3 and 6A12, which recognized β-casein in its denatured and native conformations by indirect ELISA (iELISA). Cross-reaction analysis by Western blot and iELISA indicated that these mAbs specifically recognized β-casein from bovine and goat milk extracts, while they did not cross-react with proteins present in other food matrixes. These highly specific mAbs enabled the development of sensitive, reliable and reproducible icELISAs to detect and quantify this milk protein allergen in food commodities. The extraction of β-casein from foodstuff was efficiently carried out at 60°C for 15 minutes, using an extraction buffer containing 1% SDS. The present study establishes a valid 1H3 based-icELISA, which allows the detection and quantification -0.29 ppm and 0.80 ppm, respectively- of small amounts of β-casein in raw and processed foods. Furthermore, we were able to detect milk contamination in incurred food samples with the same sensitivity as a commercial sandwich ELISA thus showing that this icELISA constitutes a reliable analytical method for control strategies in food industry and allergy prevention.

## Introduction

Food allergy is an adverse immune-mediated response that occurs reproducibly on exposure to a given food [1]. It stands as a growing public health concern due to its increasing prevalence and life-threatening potential, only mitigated by avoidance of allergen-containing foods. Food allergy affects up to 5% of the adult population and 8% of young children [2]. The members of the "big eight" group of allergenic foods -milk, egg, wheat, peanuts, tree nuts, soy, crustaceans and fish- are responsible for 90% of the food allergic reactions [3]. The Food Allergen Labeling and Consumer Protection Act (FALCPA) of 2004 mandated the declaration of these eight major food allergens on labels of packaged foods sold in the United States.

Cow's milk (milk from now on) protein allergy (CMPA) is the most common childhood food allergy, with a prevalence within 2% to 7.5% [4,5]. Although most children outgrow the disease by 3 years of age, CMPA does sometimes persist or can newly develop in adulthood. It has been suggested that CMPA in adults, arising as gastrointestinal reactions, may be more common than formerly thought [6,7]. CMPA can be characterized as any adverse reaction mediated by immunological mechanisms to one or several proteins found in milk [8]. CMPA presents a multiplicity and degrees of symptoms, which arise from the gut, skin and respiratory tract. The clinical manifestations can be immediate or delayed, and may operate separate or together. The immediate reactions, which are mainly IgE-dependent, include cutaneous reactions with urticaria and edema, respiratory episodes, gastrointestinal (GI) distress including vomiting, diarrhea and bloody stools, and anaphylaxis. The delayed-onset phenomenon, which takes place upon T-cell dependent mechanisms from 1 h to several days after ingestion of milk, is also characterized by cutaneous, respiratory and GI symptoms, including disorders like atopic dermatitis, milk-induced pulmonary disease, chronic diarrhea, and gastroesophageal reflux disease [9–12]. The anaphylactic shock is a particularly severe symptom, noted in 15% of CMPA patients, which may result in death. CMPA's treatment is complete avoidance of milk proteins. Symptoms are mitigated upon dietary elimination of the responsible antigen and, ideally, a brief re-challenge should be carried out to confirm that symptoms recur [1]. To accomplish total exclusion of milk proteins in their diet, patients suffering from CMPA have to consider three types of potential cross-reactivity or cross-contamination: 1) between milks from different animal species; 2) between milk and other foods; 3) between foods or drinks in the manufacturing process. First, allergies to milk proteins of other non-bovine mammals have been reported due to cross-reactivity between bovine milk proteins and their counterpart in other species, such as sheep, goat and buffalo, sharing 91%, 90% and 96% of sequence identity with bovine β-casein, respectively [1,13]. Second, CMPA patients cannot consume milk's derivatives -cheese, yoghurt, milk-based ice-cream, etc.- and should be careful with possible cross-reactivity between milk and meat or animal dander [14]. Third, and finally, cross-contamination in food industry during the manufacturing process is well known as a result of inadequate cleaning of industrial equipment between production changes. For instance, traces of milk can be found in dark chocolate, hamburger steak, hot dogs, sausages and natural flavorings. In addition, processing aids used in food and drink production, which are not expected to be contained in the final product, are nevertheless frequently found. For example, the use of potassium caseinate as a fining agent in wines can leave traces of casein in the final product, implying a potential threat to an allergic consumer [15]. Therefore, standardized manufacturing practices and reliable food specifications to protect sensitive consumers from "hidden allergens" is encouraged, yet is generally voluntary and not properly regulated [16,17].

During the processing of food components, allergenicity of proteins may be altered by different methods, such as heating, pressurization or sterilization [18]. As far as it concerns milk, the allergenic properties of its proteins may increase, decrease or remain unchanged upon processing. The molecular basis of the alterations in the allergenic activity is the inactivation or destruction of epitope structures, formation of new epitopes (neotopes), or greater access to hidden epitopes by denaturation of the native allergen (cryptotopes). In particular, heating processes can only modify conformational epitopes, since they might lose their binding capacity to specific IgEs, but linear epitopes preserve their allergenicity upon heating [16,17,19]. It has been reported that caseins, unlike whey proteins, are considerably resistant to heat treatment and maintain their allergenic properties after pasteurization (70–80°C for 15–20 sec) and sterilization (>100°C for 2–4 sec) processes [16,20–22].

Current methods to detect allergens in food rely on markers associated to the allergen-containing ingredient (i.e., proteins, peptides, DNA). Protein-based analytical methods include enzyme-linked immunosorbent assay (ELISA), immunochromatography, Western blotting and Mass Spectrometry, whereas DNA-based analytical methods mostly rely on polymerase chain reaction (PCR) assays [23]. From these methods, ELISA is the most commonly used for the detection of allergenic substances in food due to its relative ease of use, high precission, sensibility and potential for standarization. Reliable detection of allergens usually constitutes a challenge, mainly due to food processing effects impacting on the three dimensional structure of the proteins contained in food. Antibodies generated to detect native allergens generally show decreased or no reactivity against the denatured protein.

Milk is considered a high nutritional protein source, supplying 32 g protein/L, approximately. It is composed of soluble and insoluble proteins, named whey proteins and caseins respectively. Whey proteins -β-lactoglobulin, α-lactoalbumin, immunoglobulins, bovine serum albumin and lactoferrin- represent 20% of the milk protein fraction, whereas the casein fraction -α-, β- and κ-caseins, and the γ-caseins derived from the hydrolysis of β-casein- constitutes 80% of the total protein. Although milk contains more than 20 proteins with allergenic potential, caseins and, to a minor extent, β-lactoglobulin, are recognized as the major allergens [16,24,25]. Caseins are found in milk as components of the casein micelle, in which thousands of molecules of α-, β- and κ-caseins aggregate through protein-protein interactions forming complexes with calcium phosphate [26]. The sequences involved in β-casein intermolecular interactions within the casein micelle are mainly the Pro- and Gln-rich regions at the C-terminus [27]. Furthermore, the high content of Pro prevents the formation of α-helixes or β-sheets on the secondary structure, thus allowing β-casein to be considered as an unfolded protein, together with the rest of caseins [26]. The high content of phosphoseryl residues predominantly found in the N-terminal end of β-casein contributes to micelle stability by providing hydrophilicity towards this end of the molecule [26]. The disposition of the aggregated caseins into amorphous micelles has been hypothesized as a way to prevent amyloid fibril formation by self-association, with κ-casein binding to the surface of growing aggregates, thus limiting the growth of the particles [26]. β-casein unfolded open structure is prone to interaction with other proteins, and thus, it is of extreme importance to perform the extraction of this allergen from processed foods with buffers that can dissociate any possible intermolecular interaction and expose its full sequence in order to perform detection. Selection of extraction buffers is a current challenge in the detection of food allergens by ELISA since denatured and aggregated proteins are less water-soluble than native proteins. Surfactants (e.g. Sodium Dodecyl Sulfate, SDS) and reductants (e.g. 2-Mercaptoethanol, 2-ME) are frequently added to the extraction buffer to help in the solubilization and extraction of these proteins [28,29]. An extraction solution containing surfactants and reductants can negatively affect the stability of the antibody-antigen complex, thus reducing accuracy in the measurement by ELISA. This is the reason why many commercial quantitative ELISA kits suppliers suggest that the extract should be diluted before measurement, sometimes difficulting the detection of the allergen [29,30].

Considering the common occurrence of milk proteins as "hidden allergens" in food products, as well as the need to use harsh extraction buffers containing surfactants to solubilize higher amounts of proteins, we aimed at obtaining monoclonal antibodies (mAbs) that would recognize β-casein, the most abundant and antigenic casein along with α_S1_-casein, independently of its conformational state (native and SDS-denatured) [31]. With this strategy in mind, we have developed and characterized two mAbs -1H3 and 6A12- that are highly specific to β-casein in its native and SDS-denatured forms. Using these mAbs we were able to develop sensitive, reliable and reproducible indirect competitive ELISAs (icELISAs). In particular, further dilution of the extracted samples, contained in 1% SDS extraction buffer, was not necessary for detection and quantification of β-casein in spiked or incurred samples by icELISA. The developed 1H3-based icELISA allows the detection and quantification of small amounts of β-casein in raw and processed foods and constitutes a reliable analytical method for contaminant control strategies, and thus, the prevention of anaphylaxis episodes.

## Materials and methods

### β-casein denaturation

Bovine β-casein (Sigma-Aldrich) was denatured in 1% SDS at 100γC for 5 min. To remove SDS, 0.1 M KCl was added and incubated at 4γC for 10 min to form the KDS precipitate that was pelleted by centrifugation at 13000 rpm for 5 min. Denatured β-casein SDS free was collected in the supernatant.

### Immunizations and hybridoma generation

All mice were obtained from our own breeding facility and were housed under specific pathogen free (SPF) conditions. 8- to 9-week-old male BALB/c mice were immunized intraperitoneally with 20 μg of denatured β-casein (Sigma-Aldrich) emulsified in complete Freund's adjuvant. Booster injections of 10 μg of denatured β-casein in incomplete Freund's adjuvant were applied 21 and 42 days after the first immunization. Based on the humoral response of a test bleed performed 7 days after the final immunization, the highest BALB/c responder -according to an indirect-enzyme-linked immunosorbent assay (iELISA) (see *indirect-enzyme-linked immunosorbent assay*)- was selected as donor of spleenocytes for hybridoma production and euthanized by cardiac puncture exsanguination under complete anesthesia. Mice were monitored daily on weekdays. There were no deaths associated to immunizations or care. Hybridomas were generated by fusion of spleen cells with Sp2/0-Ag14 myeloma cells as described previously [32]. Screening of positive secreting hybridomas was carried out by iELISAs. A test sample was considered positive if the ratio (T/C) of the OD value in the test well (T) to that of the negative control well (C) was ≥2.1. We obtained 21.5% of positive hybridoma populations to denatured and native β-casein. Two hybridomas expressing high affinity mAbs against denatured and native β-casein (1H3 and 6A12) were selected and cloned twice. A completed ARRIVE guidelines checklist is included in S1 Checklist.

### Monoclonal antibodies purification

Monoclonal antibodies were purified by protein G affinity chromatography (mAbia Labs, Argentina) from hybridoma culture supernatants.

### Food matrixes extraction

One gram of homogeneized food sample was resuspended in 10 ml of extraction buffer (1% SDS) and incubated at 60°C for 15 min with vigorous vortexing every 5 min. When indicated, the food matrix was supplemented with 10 or 50 μl of 0.1 μg/μl native β-casein (Sigma-Aldrich), or with 1% 2-mercaptoethanol. The sample was centrifuged at 13000 rpm for 5 min at RT to obtain the extract in the supernatant. If neccesary, the supernatant was filtered through a Whatman Mini-UniPrep Syringeless 0.45 μm filter device (GE Healthcare). Recovery tests were carried out by competitive iELISA (see *competitive indirect-enzyme-linked immunosorbent assay*) on the same day of the extraction of the food matrixes.

### Indirect enzyme-linked immunosorbent assay

Microtiter plates (Nunc Maxisorp 96-well ELISA plates) were coated with 100 μl of denatured β-casein (Sigma-Aldrich, 10 μg/well) for hybridoma screening, or with 100 μl of the indicated concentration of native or denatured β-casein (Sigma-Aldrich) or food protein extract in coating buffer (0.1 M Na_2_HPO_4_ buffer pH 9.5) for 18 h at 4°C. Following incubation in blocking buffer [2% bovine serum albumin (BSA) in TBS] for 1 h at 37°C, the plates were further incubated with 0.1 μg/ml of the indicated purified mAb for 1 h at RT in blocking buffer. Following four washing steps in TBS Tween-20 0.05%, plates were further incubated for 1 h at RT with HRP goat anti-mouse IgG secondary antibody (Sigma-Aldrich) at a 1:6000 dilution in blocking buffer. Finally, plates were washed four times in TBS Tween-20 0.05% and after incubation with the substrate [0.3% H_2_O_2_, 0.1% 3,3',5,5'-tetramethylbenzidine (TMB) in 0.1 M citric acid pH 5] for 5 to 20 min at RT, the reaction was stopped with 0.2 M H_2_SO_4_. The absorbance at 450 nm was measured with a FilterMax F5 Multi-Mode microplate reader (Molecular Devices).

### Isotyping of immunoglobulins

The isotypes of the mAbs were determined from hybridoma culture supernatants with the Mouse Ig Isotyping Ready-SET-Go! kit (Affymetrix, eBioscience) according to the manufacturer's instructions.

### Western blotting

Food protein extracts, purified β-casein (Sigma-Aldrich) and fresh milk -UHT skim milk (milk), raw milk (a kind gift from Gabriela Rodriguez, INTI lacteos, Buenos Aires, Argentina), 10% skim milk powder (milk powder) and goat milk-, were resolved on 10% SDS-PAGE. After transfer to a nitrocellulose membrane (Hybond-ECL, GE Healthcare), analysis by immunoblotting was performed using 0.02 μg/ml 1H3 or 0.2 μg/ml 6A12 purified monoclonal antibodies in blocking buffer (5% BSA in TBS). Bound antibodies were recognized with an HRP goat anti-mouse IgG secondary antibody (Sigma-Aldrich) at a 1:6000 dilution in blocking buffer, or with Alexa Fluor 680 goat anti-mouse IgG secondary antibody (Invitrogen) at a 1:20000 dilution in blocking buffer. The signal was visualized with enhanced chemiluminiscence reagent (GE Healthcare) and CL-XPosure Films (Thermo Scientific), or with an Oddysey Infrared Imager (Li-Cor). Densitometric analysis was performed using the Li-Cor Image Studio Lite software.

### Competitive indirect enzyme-linked immunosorbent assay

Microtiter plates (Nunc Maxisorp 96-well ELISA plates) were coated with 100 μl of denatured β-casein (Sigma-Aldrich, 0.05 μg/well) in coating buffer (0.1 M Na_2_HPO_4_ buffer pH 9.5) for 18 h at 4°C. Plates were incubated in blocking buffer (2% BSA in TBS) for 1 h at 37°C. Denatured β-casein -serially diluted in blocking buffer-, spiked samples or incurred samples in a final volume of 50 μl were mixed with 50 μl of 1H3 or 6A12 mAbs at 0.1 μg/ml in blocking buffer, giving a final dilution of the sample of 1:20. Mixed samples were preincubated for 1 h at RT and then added to the wells. Detection of antibodies with secondary antibody, reaction development for 15 min at RT and absorbance measurement were carried out as described in the *indirect-enzyme-linked immunosorbent assay*.

### Sandwich enzyme-linked immunosorbent assay

The commercial RIDASCREEN® FAST Casein (R4612) kit from R-biopharm was used for β-casein quantification on incurred samples. Briefly, 1 g of homogeneized food sample was resuspended in 4 ml of Extractor 2 buffer, mixed vigorously and incubated at 100°C for 10 min. After the cooked sample was cooled down shortly, 16 ml of heated (60°C) Allergen Extraction Buffer containing Additive 1 were added, and it was mixed vigourously and incubated at 60°C for 10 min. Next, the sample was cooled down in an ice-water bath and centrifuged at 2500 *g* for 10 min at RT to obtain the extract in the supernatant. This supernatant was further centrifuged at 13000 rpm for 10 min at RT. The particle free supernatant was diluted 1:5 with Allergen Extraction Buffer, reaching a final dilution of the sample of 1:100, and immediately assayed in duplicate according to the manufacturer's instructions.

### Statistical analysis

The software GraphPad Prism 5.0 (GraphPad Software, La Jolla, CA, USA) was used for the non linear fitting of the standard curves to a 4 parameter logistic regression and for the calculation of the IC50 parameter. The CR (cross-reactivity) values were calculated as (IC50 of the antigen for which the mAb was developed/IC50 of the antigen analyzed)*100. Reproducibility (intra-plate variability) was assessed by measuring the standard curve six times for the same denatured β-casein sample on the same ELISA plate. Repeatability (inter-plate variability) was calculated by measuring the standard curve for different denatured β-casein samples on two different ELISA plates on different days. The CV (coefficient of variation) was estimated as follows: standard deviation/mean*100. The limit of detection (LOD) and the limit of quantification (LOQ) were calculated by the following formulas: LOD = mean blank samples±3*SD blank samples and LOQ = mean blank samples±10*SD blank samples.

### Ethics statement

The protocol of animal immunization followed in this study was approved by the Committee on the Ethics of Animal Experiments of the Universidad Nacional de San Martín (Resolution No. 03/2017), according to the recommendations of the Guide for the Care and Use of Laboratory Animals of the National Institutes of Health.

## Results

### Production and characterization of anti-β-casein monoclonal antibodies

In order to obtain hybridomas secreting antibodies that would specifically bind to β-casein, mice were immunized intraperitoneally with SDS-denatured β-casein (β-casein D). Hybridomas were produced by fusion of spleen cells from the immunized mice and myeloma cells. Screening by indirect ELISA (iELISA) was performed using β-casein D and native β-casein (β-casein N) in independent assays, in order to select hybridoma clones that would recognize β-casein with or without SDS treatment. Two hybridomas expressing high affinity mAbs against β-casein N and D (1H3 and 6A12) were selected and cloned twice. Both isotypes were determined to be IgG1 with lambda light chains (Table 1).

**Table 1:** Isotypes of 1H3 and 6A12 mAbs.

	IgG1	IgG2a	IgG2b	IgG3	IgA	IgM	Lambda	Kappa
1H3*	**2.49**	0.06	0.06	0.06	0.05	0.06	**1.61**	0.06
6A12*	**2.25**	0.05	0.05	0.05	0.05	0.10	**1.55**	0.08

*The isotypes of 1H3 and 6A12 mAbs were determined with the Mouse Ig Isotyping Ready-SET-Go! sandwich ELISA kit (Affymetrix, eBioscience).

**Fig 1:**
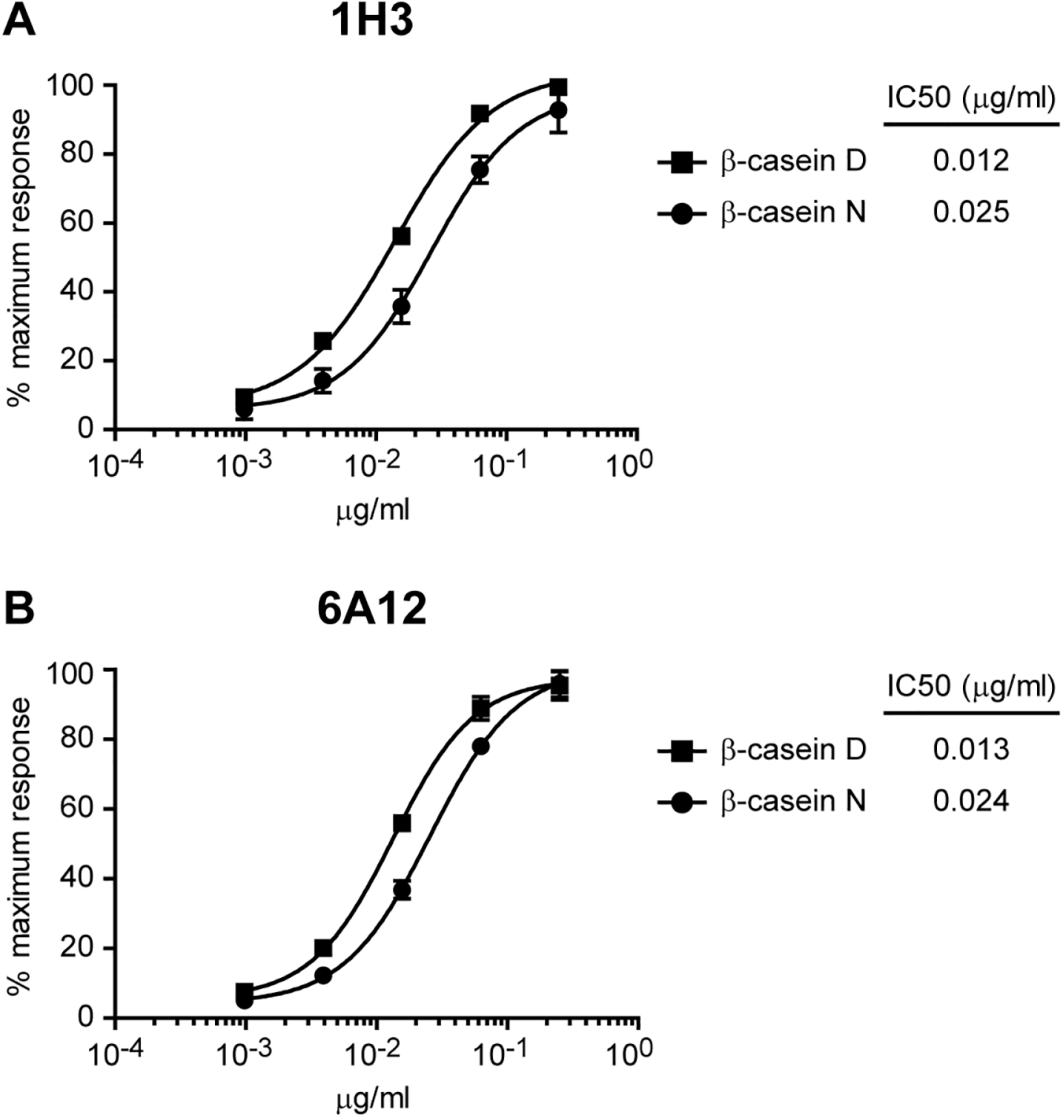
Relative affinity of 1H3 and 6A12 mAbs for denatured or native β-casein. Standard curves of denatured β-casein (β-casein D) or native β-casein (β-casein N) detected by iELISA with 1H3 **(A)** or 6A12 **(B)** mAbs. Each point of the curve represents mean±SD of three sample replicates. IC50 values of the mAbs are indicated.

Given that the developed mAbs were designed to recognize β-casein N and D, we studied their relative sensitivity for both antigens. Different concentrations of both β-casein samples were immobilized and detected with the mAbs by iELISA (Fig 1). The relative affinity of each mAb for the antigen was calculated as the concentration of the antigen conferring a 50% reduction of the peak signal in the ELISA (IC50). 1H3 and 6A12 exhibited two times higher sensitivity for β-casein D than for β-casein N (Fig 1). These results suggest that 1H3 and 6A12 mAbs recognize partially exposed epitopes on β-casein N surface, and that these epitopes become fully available for mAb recognition upon β-casein denaturation with SDS. This result implies that the α-helical secondary structure proposed for β-casein [33] is affected by SDS. Alternatively, SDS treatment might be preventing β-casein self association ocurring in the native protein [34]; this self-association phenomena might offer slight steric hindrance, preventing full interaction of the mAbs with the protein.

### Protein extraction and specificity of 1H3 and 6A12 monoclonal antibodies

**Fig 2:**
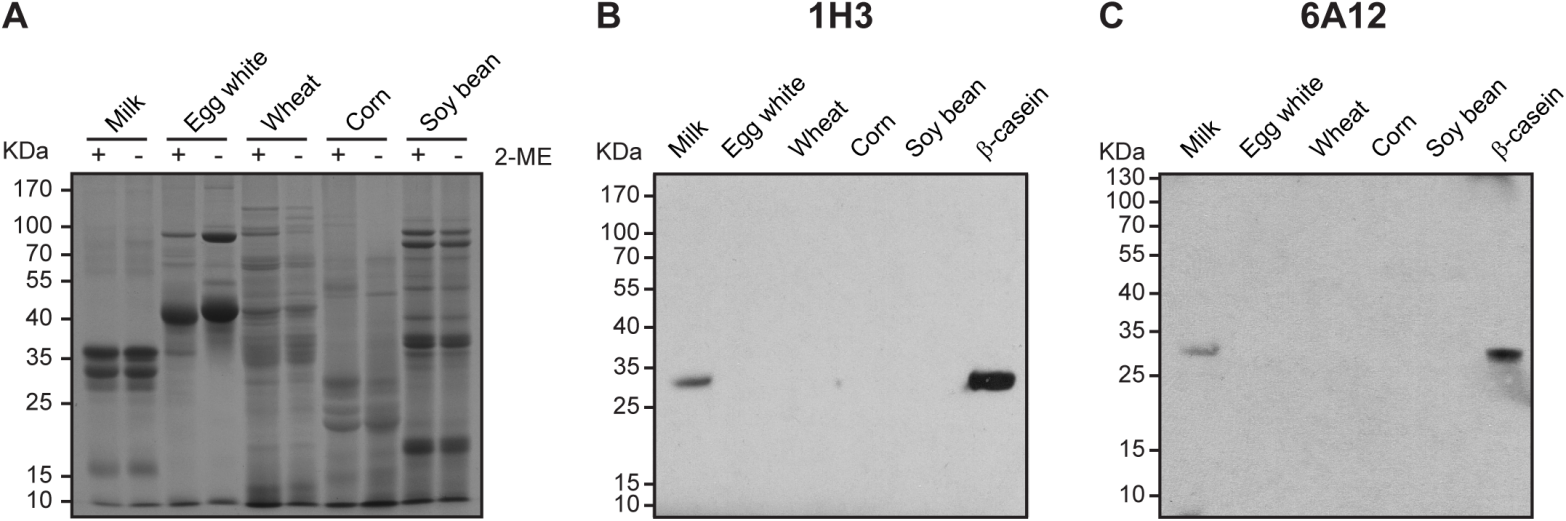
Western blot analysis of food protein extracts with 1H3 and 6A12 mAbs. **(A)** SDS-PAGE analysis of 10 μg of milk, egg white, wheat, corn or soy bean protein samples extracted with 1% SDS and 1% 2-ME (+) **(A)** or 1% SDS (-) extraction buffer **(A-C)** at 60°C for 15 min by Coomassie brilliant blue staining **(A)** or by immunoblot using 1H3 **(B)** or 6A12 **(C)** mAbs. In **(B,C)** purified bovine β-casein (2 μg) was loaded as a positive control. The position of the molecular mass standards is indicated on the left.

In a previous work, Parker and co-workers [30] compared the extractability of food allergens from different matrixes using commercial extraction buffers from several vendors. They verified that an extraction buffer containing 1% SDS and 1% 2-mercaptoethanol (2-ME) gave the highest recovery across all monitored allergens [29]. However, the reducing agent 2-ME has undesirable characteristics such as unpleasant odor, toxicity and the need of handling in a fume hood [35,36]. Because of this, we sought to carry out protein extraction from four food matrixes that are present in most food commodities: egg white, wheat, corn and soy bean, without 2-ME, and applying heat (60°C for 15 min) to the food sample resuspended in 1% SDS extraction buffer. In the presence or absence of 2-ME in the extraction buffer we obtained similar protein patterns visualized by SDS-PAGE (Fig 2A), as well as similar concentrations of extracted proteins for all the food matrixes analyzed (S1 Table). Hence, the extractions of food samples in this study were carried out in 1% SDS extraction buffer at 60°C for 15 min.

**Fig 3:**
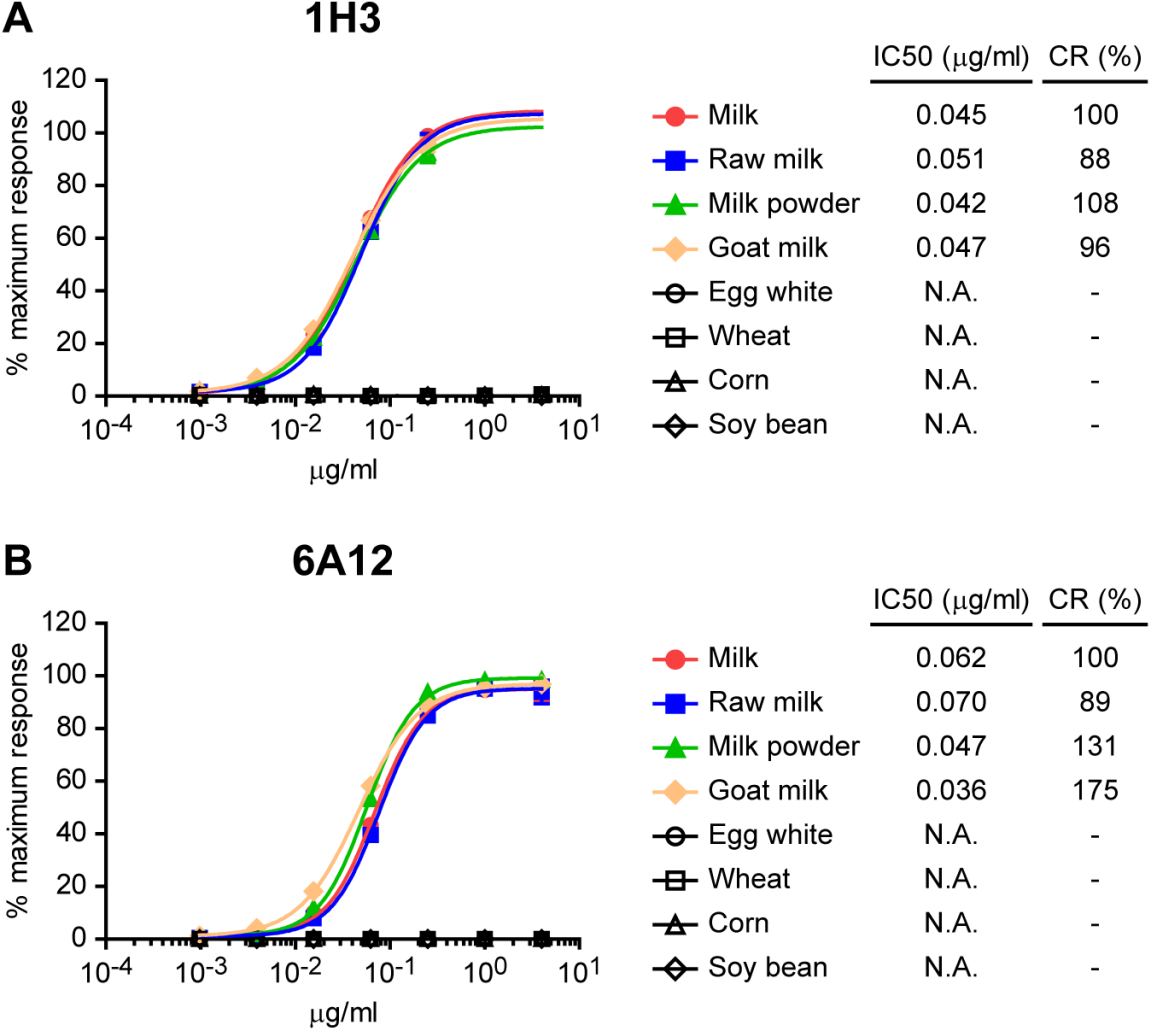
Comparative reactivity of food protein extracts by indirect ELISA with 1H3 and 6A12 mAbs. Standard curves of protein extracts (SDS- and heat-treated) from milk (UHT skim milk), raw milk, milk powder (10% skim milk powder), goat milk, egg white, wheat, corn and soy bean detected by iELISA with 1H3 **(A)** or 6A12 **(B)** mAbs. Each point of the curve represents mean±SEM of at least three sample replicates. IC50 and CR values of the mAbs are indicated. N.A.: not applicable.

To test the specificity of the developed mAbs, we first evaluated their reactivity by Western blot assays against whole bovine milk, which also contains α- and κ-caseins as well as β-lactoglobulin. Neither mAb cross-reacted with α- or κ-caseins or with β-lactoglobulin milk protein allergens (S1 Fig). Next, we assessed 1H3 and 6A12 reactivity against the previously extracted food commodities. Immunoblot analysis revealed that 1H3 and 6A12 mAbs could specifically detect bovine β-casein, while there was no recognition of proteins in the other food extracts (Fig 2B and 2C). To further obtain quantitative data about the capacity of 1H3 and 6A12 mAbs to detect β-casein from different sources, we performed an iELISA with extracted samples of milk (UHT skim milk), raw milk, milk powder (10% skim milk powder), goat milk, egg white, wheat, corn and soy bean (Fig 3). The assay proved to be highly specific for all samples of bovine milk, and also for goat milk, as was expected, given the high sequence identity between bovine and caprine β-casein [1]. No signal was observed in extracts containing egg white, wheat, corn or soy bean, as expected. Both mAbs detected raw milk with lower sensitivity than milk, while milk powder was detected with higher sensitivity. These results are consistent with the lower or higher proportion of β-casein content these milk samples have (S2 Fig). In particular, 6A12 mAb showed a higher sensitivity for goat milk than 1H3 mAb. This behaviour may be a consequence of the higher proportion of β-casein contained in goat milk, as shown in S2 Fig and as described in previous work [37], and possibly to a reduced affinity of 1H3 mAb towards caprine β-casein. Taken together, these results indicate that the developed 1H3 and 6A12 mAbs response is specifically directed towards bovine β-casein antigen, as well as to its counterpart in goat milk. Furthermore, we show that the developed mAbs can recognize bovine β-casein from different sources using an extraction method based on a surfactant (SDS) and heat (60°C) treatment.

### Development of an indirect competitive enzyme-linked immunosorbent assay using 1H3 and 6A12 monoclonal antibodies

**Fig 4:**
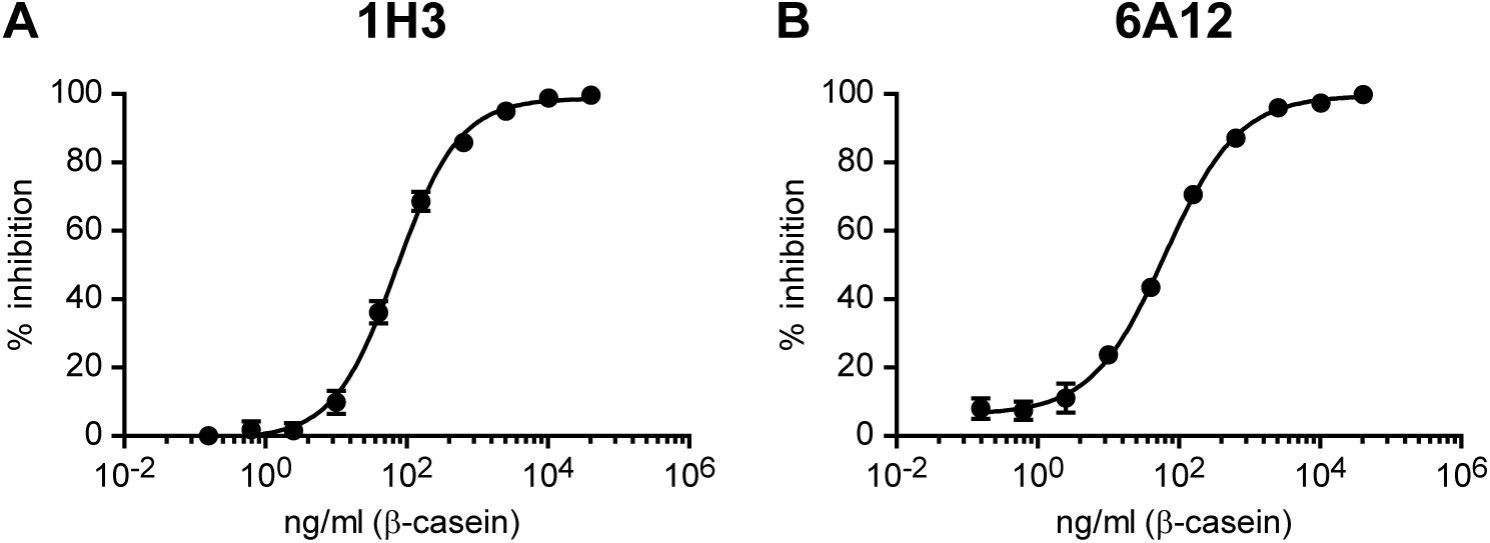
Standard curves for the detection of β-casein by indirect competitive ELISA with 1H3 or 6A12 mAbs. Standard curves of the icELISA to detect denatured β-casein using 1H3 **(A)** or 6A12 **(B)** mAbs, calculated with a 4 parameter logistic regression fitting with the following equation: Y = Bottom + (Top-Bottom)/(1+10^((LogEC50-X)*Hill slope)), where Bottom = -0.84, Top = 98.6, EC50 = 71.75 and Hill slope = 0.98 parameters were obtained for 1H3 mAb **(A)** and Bottom = 6.21, Top = 99.5, EC50 = 63.45 and Hill slope = 0.83 parameters were obtained for 6A12 mAb **(B)**. Each point of the curve represents mean±SD of six sample replicates.

Allergen detection in foodstuff requires the solubilization of the food particles in order to achieve maximum protein extraction. Ideally, a low ratio of food weight/extraction buffer volume will achieve this. If SDS is the surfactant of choice for the extraction buffer in sandwich ELISA, it might be needed to apply further dilution of the extracted solution [29]. This dilution steps will render a low concentration of the extracted proteins into the sample. Taking these considerations in mind, we tested whether an indirect competitive ELISA (icELISA) could be suitable to be used with raw extracted samples containing SDS. We performed several icELISAs to find the optimal assay conditions. A β-casein D concentration of 0.5 μg/ml fixed on the plate and a preincubation of 0.05 μg/ml 1H3 or 6A12 mAb with the sample for 1 h at room temperature were found to be optimum (data not shown). The standard curves for the detection of β-casein D by icELISA with 1H3 or 6A12 mAbs under the established conditions showed a good correlation to the data (R^2^ = 0.99). The β-casein D concentration that inhibited 50% total binding of the mAb (IC50) in the standard curve of the icELISA was 75.05 ng/ml for 1H3 and 54.73 ng/ml for 6A12 (Fig 4). The repeatability and reproducibility of the method were estimated from several standard curves carried out on the same ELISA plate (intraassay) or on different ELISA plates (interassay) respectively (Table 2). For the β-casein D standards between 40 and 2560 ng/ml, we determined an intraassay CV of 0.47–9.10% and 0.41–4.71% and an interassay CV of 0.37–6.33% and 1.38–5.93% for 1H3 and 6A12 mAbs respectively. These results show that our mAb-based icELISA meet the requirements of intraassay and interassay CV for procedures used in detection and quantitation of allergens in food [38].

**Table 2:** Intraassay and interassay variation of denatured β-casein standards analyzed by indirect competitive ELISA with 1H3 or 6A12 mAbs.

	1H3				6A12			
	Intraassay (n = 6)		Interassay (n = 2)		Intraassay (n = 6)		Interassay (n = 2)	
β-casein (ng/ml)	Inhibition (%)*	CV (%)	Inhibition (%)*	CV (%)	Inhibition (%)*	CV (%)	Inhibition (%)*	CV (%)
**40**	36.11±3.29	9.10	36.02±0.13	0.37	43.44±2.05	4.71	42.19±1.76	4.18
**160**	68.57±2.76	4.02	65.63±4.15	6.33	70.56±0.78	1.11	67.71±4.02	5.93
**640**	85.80±1.26	1.47	84.65±1.63	1.92	87.03±0.48	0.55	85.19±2.60	3.05
**2560**	94.94±0.45	0.47	95.32±0.54	0.56	95.97±0.39	0.41	95.05±1.31	1.38

*mean±SD.

**Fig 5:**
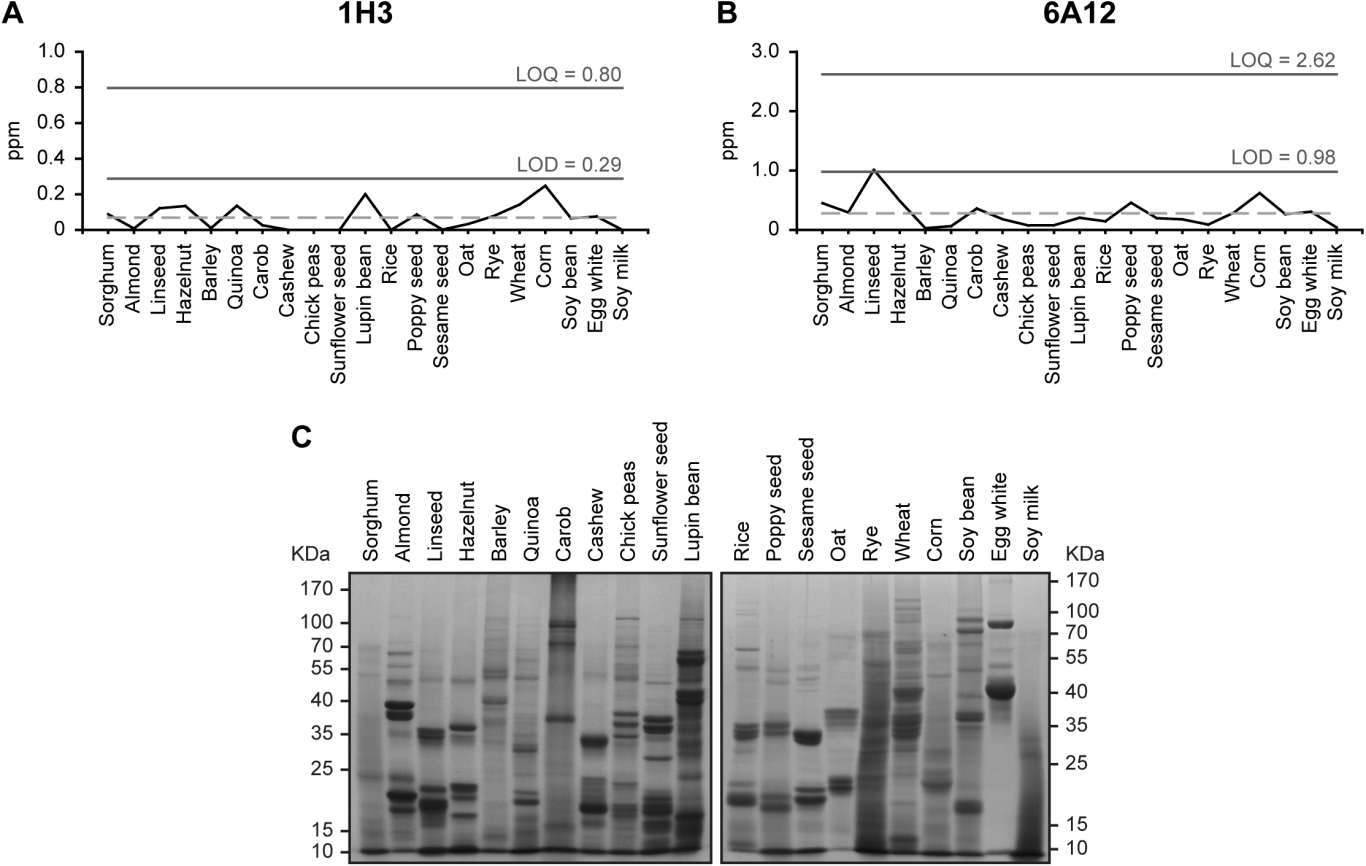
Determination of the limits of detection and quantification for the indirect competitive ELISA to detect β-casein using 1H3 or 6A12 mAbs. **(A-B)** The limit of detection (LOD) and the limit of quantification (LOQ) for the detection of denatured β-casein by icELISA with 1H3 **(A)** or 6A12 **(B)** mAbs were obtained measuring the concentration of the 21 listed food matrix samples (blank samples) and calculated by the following formulas: LOD = mean blank samples±3*SD blank samples, and LOQ = mean blank samples±10*SD blank samples. The dotted line represents the mean of the blank samples analyzed. **(C)** Coomassie brilliant blue-stained SDS-PAGE analysis of the 21 food protein extracts used for LOD and LOQ determinations. The position of the molecular mass standards is indicated.

The resulting high-sensitivity 1H3-based icELISA had a limit of detection (LOD) of 0.29 ppm and a limit of quantification (LOQ) of 0.80 ppm, while the 6A12-based icELISA showed 0.98 and 2.62 ppm for LOD and LOQ, respectively (Fig 5A and 5B). These parameters were determined upon extraction of total proteins from twenty-one blank samples (Fig 5C) [38]. Given that we aimed to quantify at least 1 ppm of β-casein in food matrixes, we performed recovery tests by icELISA with 1H3 mAb. To this purpose, egg white, wheat, corn and soy bean samples spiked with 1 or 5 ppm of β-casein N were extracted and analyzed by 1H3-based icELISA without the need of further diluting the extract (Table 3), testing whether a raw extract containing 1% SDS would be suitable for use as a sample for this assay. The average recoveries at the two supplemented levels varied from 81.9 to 118.5% and the CV ranged from 2.13 to 9.21% for all the food samples tested. These data indicate that the icELISA based on 1H3 mAb is sensitive, reliable and reproducible. Besides, it should be noted that the developed icELISA allowed the detection and quantification of β-casein D from food matrixes that were formerly spiked with β-casein N and extracted with heat treatment in the presence of 1% SDS. These results suggest that 1H3 mAb is highly specific for β-casein D, as well as for any remaining β-casein N following extraction, and that its affinity for the antigen is strong enough to circumvent any disturbance in the antibody-antigen complex caused by the surfactant agent contained in the extraction buffer.

**Table 3:** Recovery analysis of egg white, wheat, corn and soy bean supplemented with β-casein by 1H3 based-indirect competitive ELISA.

	Spiked level (ppm)	n	Mean±SD (ppm)	Recovery (%)	CV (%)
**Egg white**	1	4	0.84**±**0.06	83.8	7.68
	5	4	4.81**±**0.15	96.3	3.02
**Wheat**	1	4	0.98**±**0.09	97.6	9.21
** **	5	4	4.10±0.32	81.9	7.71
**Corn**	1	4	1.17**±**0.08	117.1	6.80
** **	5	4	5.92**±**0.29	118.5	4.88
**Soy bean**	1	3	1.09**±**0.02	109.8	2.13
** **	5	4	4.10**±**0.24	82.0	5.82

Finally, we tested whether the 1H3-based icELISA could detect and quantify β-casein from incurred samples. To this purpose, three different foodstuff from the market were analyzed: (1) milk cookies, which declared to contain milk powder; (2) fudge chocolate chip cookies, that specified they have been elaborated on an equipment that processes milk; and (3) stuffed chocolate cookies, which didn't declare to contain milk. First of all, we compared β-casein extractability from incurred samples with a 1% SDS extraction buffer with or without 2-ME, in order to confirm that our extraction buffer is as efficient as a 2-ME containing extraction buffer to extract incurred samples. Immunoblot analysis with 1H3 mAb revealed that β-casein can be equally extracted from milk cookies and fudge chocolate chip cookies in the presence or absence of 2-ME in the extraction buffer (S3 Fig). Therefore, we proceeded to extract the incurred samples with our 1% SDS extraction buffer and analyzed their β-casein content through the 1H3-based icELISA. It should be noted that the minimum dilution for our developed icELISA is 1:20, given that 1 g of food matrix is resuspended in 10 ml of extraction buffer for extraction, and that this extracted sample is further diluted 1:2 when it is incubated with the mAb. For milk cookies and fudge chocolate chip cookies it was necessary to further dilute the samples to a final dilution of 1:64000 and 1:2000 respectively to be in the linear range of the curve. We obtained a β-casein content of 5513 ppm and 228 ppm for the milk cookies and fudge chocolate chip cookies respectively. On the other hand, we were able to detect β-casein in the stuffed chocolate cookies, although we were not able to quantify it because the obtained ppm values were under the LOQ (Table 4). Given that the analyzed incurred samples do not declare the amount of contained milk -either as an ingredient, or as a contaminant-, we compared our results with the results from the R-Biopharm RIDASCREEN® FAST Casein sandwich ELISA. We analyzed the same incurred samples, but using the extraction buffer and indications provided by the R-Biopharm commercial kit. It is worth noting that the minimum dilution for this sandwich ELISA is 1:100, since 1 g of food sample is resuspended in 20 ml of extraction buffer for extraction, and the extracted sample is further diluted 1:5 before its incubation in the plate. Using this kit we obtained 9625 ppm of β-casein in the milk cookies (final dilution 1:64000) and 144 ppm of β-casein in the fudge chocolate chip cookies (final dilution 1:1000). We were not able to detect β-casein in the stuffed chocolate cookies as we obtained a ppm value that was lower than the LOD (Table 4). Taken together, these results indicate that the 1H3-based icELISA can perform as good as, or better than, a commercial kit of one of the leader brands in the market regarding incurred samples. The β-casein content of the milk cookies and the fudge chocolate chip cookies quantified by the icELISA or the commercial sandwich ELISA are in the same order of magnitude. Besides, the 1H3-based icELISA performance for the quantification of β-casein from the fudge chocolate chip cookies was superior than the R-Biopharm sandwich ELISA given that the former was able to quantify β-casein content from a 1:2000 dilution, yet the latter was under the LOQ for the same dilution. This higher sensitivity of the icELISA was also noticed in the analysis of the stuffed chocolate cookies. Using the minimum sample dilution for each ELISA, the 1H3-based icELISA was able to detect β-casein, though the commercial sandwich ELISA could not detect this milk protein allergen.

**Table 4:** Comparison of β-casein content on incurred samples from the market by 1H3 based-indirect competitive ELISA and by R-biopharm RIDASCREEN® FAST Casein sandwich ELISA.

		1H3 icELISA		RIDASCREEN® FAST Casein	
Sample	Milk declared	Dilution	ppm*	Dilution	ppm*
**Milk cookies**	milk powder	1:64000	5513	1:64000	9625
**Fudge chocolate chip cookies**	Produced on equipment that processes milk	1:2000	228	1:2000	<LOQ
		1:1000	203	1:1000	144
**Stuffed chocolate cookies**	n.s.	1:20	<LOQ	1:100	<LOD

*ppm values were calculated considering the sample dilution.

n.s.: not specified.

## Discussion

CMPA is usually the first allergy to manifest itself as cow milk proteins constitute the first source of antigens encountered in large quantities during infancy [39,40]. Although CMPA is the most frequent childhood food allergy, its occurrence in adults-which is more common than formerly thought-, shows more severe symptoms than in children in response to lower doses of the allergenic protein [4–7,41]. Besides, CMPA is considered an important initial link in the allergic disease family, since it is related with an increased risk of developing other allergic disorders, such as allergic asthma, eczema or rhinoconjunctivitis among others [42]. Thus, complete dietary elimination of milk proteins is highly encouraged in CMPA's patients. To this task, patients suffering from CMPA have to avoid the consumption of milk's derivatives and milk from other animal sources, due to potential cross-reactivities between cow milk proteins and their counterpart in other species. In addition, they should be aware of any "hidden allergens" in foodstuff, which are the result of cross-contamination in food industry during the manufacturing process because of the inadequate cleaning of industrial equipment between production changes, or the incomplete elimination of aids to the processing of food and drink from the final product [1,13,16,17]. Therefore, food industry and regulatory bodies require standardized manufacturing practices and reliable advisory labeling to safeguard the food-allergic population from "hidden allergens" [16]. In addition to allergen cross-contamination during manufacture practices, the problem of alteration of allergenic potencies is present in processed foods. The allergenic activity may be unchanged, decreased or even increased upon food processing. In regard to milk proteins, it has been reported that caseins antigenicity remains unaffected upon heat treatment [16,20–22]. In view of the common occurrence of milk proteins as "hidden allergens" in food products, as well as the caseins heat-stability properties, we aimed to obtain mAbs against native and denatured β-casein, one of the most abundant and antigenic caseins [31], in order to develop an icELISA to detect and quantify traces of this milk allergen in raw and processed foodstuffs.

Immunochemical based-analytical methods, particularly ELISA, are the most commonly applied for the detection of allergens in food. The manner in which the food has been processed can mask or alter allergenic proteins markers, thereby, food manufacture poses a significant challenge in the development of reliable methods for allergen detection and quantification [30]. The overall performance of an ELISA-based method for the detection of food allergens relies on two parameters: the efficiency of the extraction of these proteins from the sample, and the efficiency with which the antibody or antibodies used in the ELISA detect the allergens in the complex mixture of the food matrix [38].

The selection of the buffer used to extract the protein from the foodstuff is a key challenge in the detection of food allergens by ELISA [30]. Surfactants and reductants are frequently added to the extraction buffer to aid in the solubilization of denatured and aggregated proteins. However, these agents may affect the antibody-antigen complex in the ELISA measurement. Hence, the dilution of the extract is suggested to avoid any potential antibody-antigen interference, yet sometimes complicating the detection of the allergen itself [29,30]. Watanabe et. al. developed an universal protein extraction protocol that consisted on the resuspension of the food sample in 1% SDS and 1% to 7% 2-ME extraction buffer, and its incubation at room temperature overnight [29]. Given the undesirable characteristics of 2-ME [36], as well as its potential interference with the antibody-antigen complex, we aimed to eliminate it from our extraction buffer. The extraction of β-casein from food matrixes was efficiently carried out through heat treatment at 60°C for 15 min, using an extraction buffer containing the surfactant agent SDS at a 1% w/v concentration. This ionic detergent is known to bind to proteins through ionic and hydrophobic interactions, solubilizing them by altering their secondary and tertiary structure [43]. This is especially relevant for an antigen such as β-casein, which is known to associate with itself or with other caseins in milk as a result of its intrinsically disordered structure [34]. We showed that the extraction efficiency -in relation to protein concentration and SDS-PAGE pattern- was similar in the presence or absence of 2-ME in the extraction buffer for all the food matrixes analyzed. It is plausible that heat treatment, together with the effect of the SDS surfactant agent, helped in the protein solubilization without the need to add any reductant agent to the extraction buffer.

In this work, we have developed and thoroughly characterized 1H3 and 6A12 mAbs against β-casein milk protein allergen. These mAbs were designed to recognize β-casein in its denatured and native conformations. As it was shown by iELISA, they show twice the affinity for β-casein D than to β-casein N. Although hybridomas populations were screened for detection of N and D β-casein, this result was expected, given that mice were immunized with β-casein D. Although β-casein displays an open structure, that is, no defined tertiary structure at all [26], it does contain secondary structure elements [33], which might be affected during SDS and heat treatment. Hence, our extraction method might be fully exposing the epitopes detected by 1H3 and 6A12 mAbs, which might be otherwise not fully accessible. Another explanation to the higher affinity for β-casein D is that β-casein N might be in the form of self-associated complexes in solution, offering steric hindrance for the interaction with mAbs.

Western blotting and the iELISA cross-reaction study indicated that 1H3 and 6A12 mAbs can specifically recognize β-casein protein in milk extracts (milk, raw milk, milk powder and goat milk), and that it does not cross-react with other proteins present in egg white, wheat, corn and soy bean extracts. Therefore, 1H3 and 6A12 were considered highly specific and sensitive against β-casein to develop an immunodetection assay to detect and quantify this milk protein allergen in raw and processed food commodities.

Sandwich ELISA is the usually preferred ELISA method for the detection and quantification of food allergens due to its lower limits of detection. As a proof of concept, we sought to test whether an icELISA would be suitable for detecting β-casein in undiluted protein extracts containing 1% SDS. Therefore, we developed icELISAs based on 1H3 or 6A12 mAbs. These assays showed an IC50 value of 75.05 ng/ml and 54.73 ng/ml for 1H3 and 6A12 mAbs, respectively. In addition, we obtained intraassay and interassay CV values lower than 10% -indicating the repeatability and reproducibility of the assays-, and a LOD of 0.29 ppm and 0.98 ppm and a LOQ of 0.80 ppm and 2.62 ppm for 1H3 and 6A12 based-icELISAs. Since we aimed to quantify at least 1 ppm of β-casein in food matrixes, the recovery tests were performed with 1H3 based-icELISA. The results of the recovery tests indicated that the icELISA based on 1H3 mAb was sensitive, reliable and reproducible. The average recoveries were between the ideal range from 80 to 120%, and CV values were lower than 10% [38]. It is important to highlight that the developed icELISA presented an acceptable recovery of β-casein from food matrixes that were formerly spiked with β-casein N and extracted with heat treatment in the presence of 1% SDS. Given that it was not neccesary to further dilute the extracted sample for measurement by icELISA, these results suggest that 1H3 mAb is highly specific for β-casein D, as well as for any remaining β-casein N following extraction, and that its affinity for the antigen is sufficiently strong to avoid any interference in the antibody-antigen complex caused by the surfactant agent contained in the extraction buffer. On the other hand, we demonstrated that the 1H3-based icELISA is a valid analytical method to quantify β-casein from incurred samples. Its performance proved to be as good as, or better than, the R-Biopharm RIDASCREEN® FAST Casein sandwich ELISA. We determined ppm values in the same order of magnitude for the β-casein content of milk cookies and fudge chocolate chip cookies by the icELISA and the commercial sandwich ELISA. Moreover, the icELISA showed a higher sensitivity than the R-Biopharm kit in the analysis of the fudge chocolate chip cookies and the stuffed chocolate cookies. This commercial kit has a LOD of 0.71 ppm and a LOQ of 2.5 ppm. We presume that its lower sensitivity is due to the need of diluting the extract 1:5 before its incubation on the plate, probably because of the presence of 2-ME in the Extractor 2 buffer used for the sample extraction. Therefore, it is important to highlight that the developed 1H3-based ELISA has the advantage of assaying food samples extracted in a 1% SDS extraction buffer that does not contain 2-ME, and that these samples can be used directly without further diluting since the surfactant does not disturb the antibody-antigen high affinity complex.

In the last years, a "reproducibility crisis" phenomena has emerged as a consequence of flaws in the reliability of antibodies. Due to deficiencies of thorough validation and characterization policies, many commercial antibodies have been demonstrated to bind more than one target [44,45]. In this context, it should be emphasized that we have carefully validated 1H3 and 6A12 mAbs against their antigens, and against antigens with the higher chances of cross-reaction. Moreover, the advantageous characteristic of mAbs versus polyclonal antibodies, which are usually included in commercially available quantitative ELISA kits, is the absence of batch-to-batch variability introduced by their production in animals. Finally, we have shown 1H3 and 6A12 mAbs to be specific, selective and reproducible in the quantitative icELISA methods we have validated [38].

In summary, mAbs highly specific to β-casein in its denatured and native conformations were developed and characterized. These mAbs enabled the development of immunodetection assays to detect and quantify this milk protein allergen in foodstuff. The present study establishes a valid icELISA, based on 1H3 mAb, which allows the detection and quantification of small amounts of β-casein in raw and processed foods -including incurred samples-, constituting a reliable analytical method for control strategies and allergy prevention.

## Supporting information

S1 Checklist Completed “The ARRIVE Guidelines Checklist” for reporting animal data in the manuscript. (PDF)

S1 Table Extraction of proteins from food allergen reference matrixes by SDS/2-ME or SDS extraction buffer at 60°C for 15 minutes. (PDF)

S1 Fig Specificity of 1H3 and 6A12 mAbs to β-casein. SDS-PAGE analysis of 1 μg of milk protein extract by immunoblot using 1H3 **(A)** or 6A12 **(B)** mAbs, or of 7 μg of milk protein extract by Coomassie brilliant blue staining **(C)**. Purified bovine β-casein (0.1 μg for the immunoblot assays or 0.7 μg for the Coomassie staining) was loaded as a positive control. The position of the molecular mass standards is indicated on the right.
(TIF)

S2 Fig Coomassie brilliant blue-stained SDS-PAGE analysis of milk proteins. SDS-PAGE analysis by Coomassie brilliant blue staining of 10 μg of fresh milk (UHT skim milk), raw milk, milk powder (10% skim milk powder) or goat milk, or 2 μg of purified bovine β-casein. The percentage of the relative β-casein content is indicated for each extract. The position of the molecular mass standards is shown on the left.
(TIF)

S3 Fig Western blot analysis of incurred samples extracts with 1H3 mAb. SDS-PAGE analysis of equal sample volumes of milk cookies and fudge chocolate chip cookies extracted with 1% SDS (-) or 1% SDS and 1% 2-ME (+) extraction buffer at 60°C for 15 min by immunoblot using 1H3 mAb. The position of the molecular mass standards is indicated on the left.
(TIF)
