# Supplementary material for: Novel sensitive monoclonal antibody based competitive enzyme-linked immunosorbent assay for the detection of raw and processed bovine beta-casein
Source: PLoS One. 2017 Jul 31;12(7):e0182447. doi: 10.1371/journal.pone.0182447 (PMC5536360; doi:10.1371/journal.pone.0182447)
Supplement: S1 Table — (PDF) [file pone.0182447.s002.pdf]

	SDS/2-ME	SDS	
	Total protein (mg)	Total protein (mg)	Protein relative to SDS/2-ME extract (%)
**Milk**	52	54	105
**Egg white**	161	173	108
**Wheat**	63	57	91
**Corn**	44	35	80
**Soy bean**	132	150	114
