# Supplementary figures and images for: Novel sensitive monoclonal antibody based competitive enzyme-linked immunosorbent assay for the detection of raw and processed bovine beta-casein
Source: PLoS One. 2017 Jul 31;12(7):e0182447. doi: 10.1371/journal.pone.0182447 (PMC5536360; doi:10.1371/journal.pone.0182447)

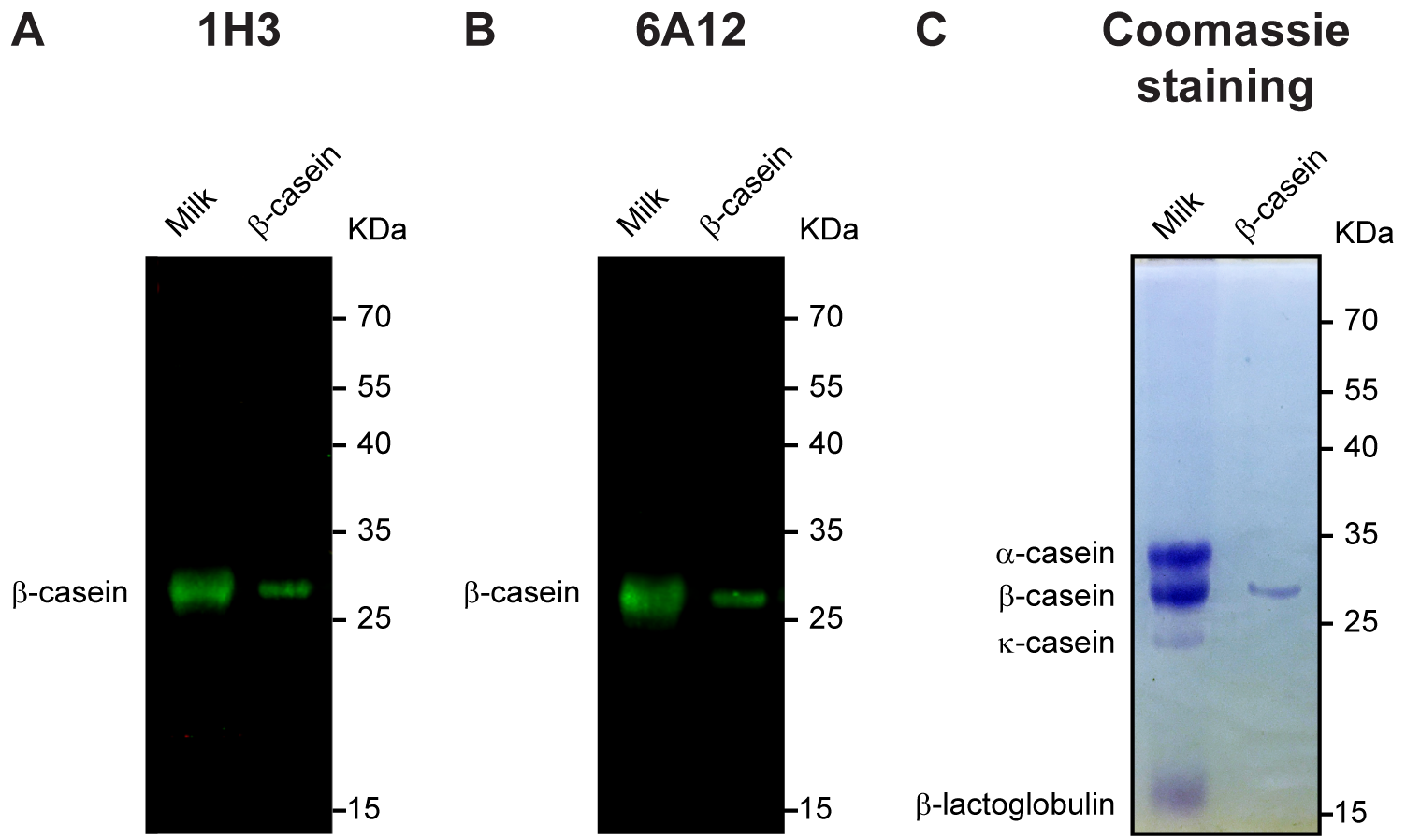

Supplement: S1 Fig — SDS-PAGE analysis of 1 μg of milk protein extract by immunoblot using 1H3 (A) or 6A12 (B) mAbs, or of 7 μg of milk protein extract by Coomassie brilliant blue staining (C). Purified bovine β-casein (0.1 μg for the immunoblot assays or 0.7 μg for the Coomassie staining) was loaded as a positive control. The position of the molecular mass standards is indicated on the right. (TIF) [file pone.0182447.s003.tif]

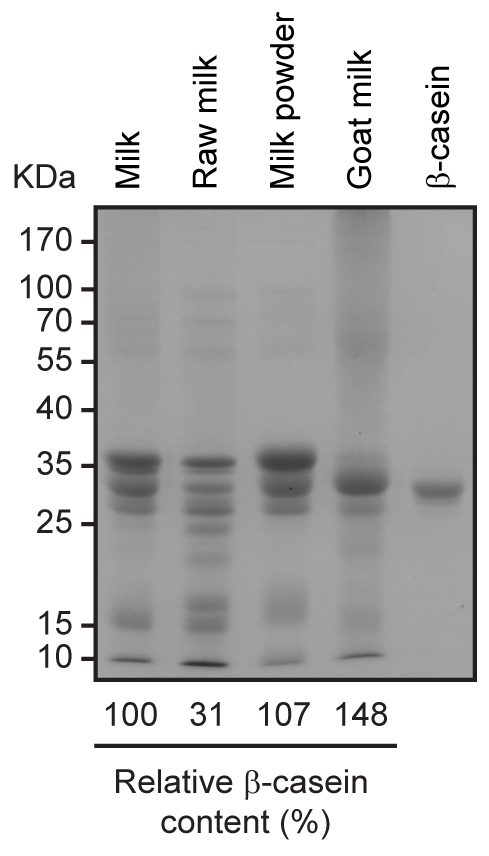

Supplement: S2 Fig — SDS-PAGE analysis by Coomassie brilliant blue staining of 10 μg of fresh milk (UHT skim milk), raw milk, milk powder (10% skim milk powder) or goat milk, or 2 μg of purified bovine β-casein. The percentage of the relative β-casein content is indicated for each extract. The position of the molecular mass standards is shown on the left. (TIF) [file pone.0182447.s004.tif]

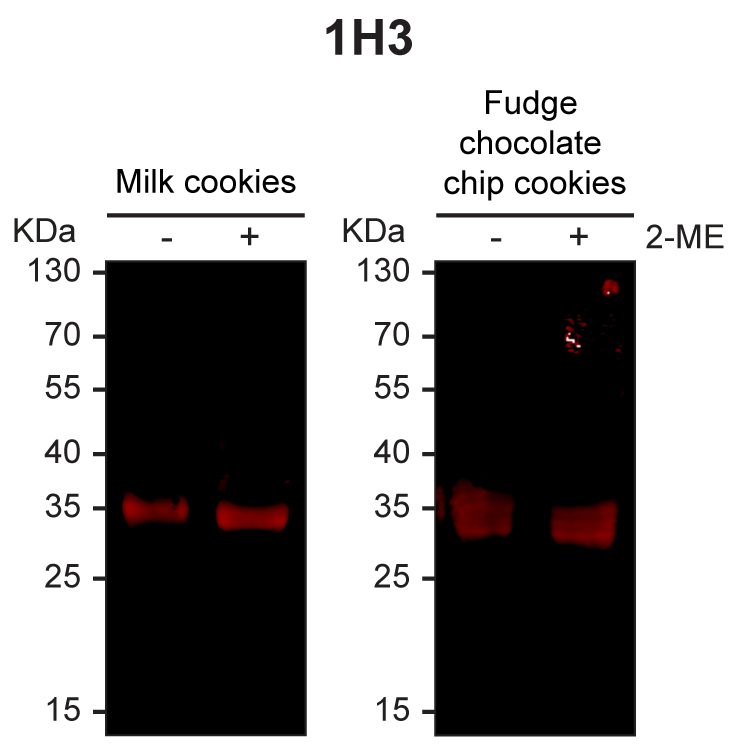

Supplement: S3 Fig — SDS-PAGE analysis of equal sample volumes of milk cookies and fudge chocolate chip cookies extracted with 1% SDS (-) or 1% SDS and 1% 2-ME (+) extraction buffer at 60°C for 15 min by immunoblot using 1H3 mAb. The position of the molecular mass standards is indicated on the left. (TIF) [file pone.0182447.s005.tif]
